# Expanded antiretroviral treatment, sexual networks, and condom use: Treatment as prevention unlikely to succeed without partner reduction among men who have sex with men in China

**DOI:** 10.1371/journal.pone.0171295

**Published:** 2017-04-13

**Authors:** Jie Lou, Peipei Hu, Han-Zhu Qian, Yuhua Ruan, Zhen Jin, Hui Xing, Yiming Shao, Sten H. Vermund

**Affiliations:** 1 Department of Mathematics, Shanghai University, Shanghai, China; 2 Vanderbilt Institute for Global Health, Vanderbilt University, Nashville, Tennessee, United States of America; 3 Division of Epidemiology, Department of Medicine, Vanderbilt University School of Medicine, Nashville, Tennessee, United States of America; 4 State Key Laboratory for Infectious Disease Prevention and Control, National Center for AIDS/STD Control and Prevention, Chinese Center for Disease Control and Prevention, Collaborative Innovation Center for Diagnosis and Treatment of Infectious Diseases, Beijing, China; 5 Complex Systems Research Center, Shanxi University, Taiyuan, China; 6 Department of Pediatrics, Vanderbilt University School of Medicine, Nashville, Tennessee, United States of America; University of New South Wales, AUSTRALIA

## Abstract

**Background:**

To project the impact of partner reduction on preventing new HIV infections among men who have sex with men (MSM) under varying conditions of enhanced HIV testing and treatment (T&T) and condom use in Beijing, China.

**Methods and findings:**

A complex network model was fitted to predict the number of new HIV infections averted from 2014 to 2023 under four scenarios of sexual behavior risk reduction (S)—*S1*: Male sexual partners decrease (reduced by a random value *m* from 1–50) while condom use increases (risk constant *p* is a random value between 0.2 and 1]); *S2*: Both sexual partners and condom use decrease (*m* 1, 50; *p* 1, 1.8); *S3*: Sexual partners reduce (*m* 1, 10) while condom use increases or decreases (*p* 0.2, 1.8); *S4*: Only MSM with ≥100 male sexual partners reduce their partners (*m* 1, 50) while condom use increases (*p* 0.2, 1).

HIV prevalence will reach 23.2% by 2023 among Beijing MSM if T&T remains at the 2013 level. The three most influential factors are: T&T coverage; partner reduction (*m*); and the background risk (*p*). Under scenarios 1–4 of sexual behavioral changes with enhanced T&T interventions, the cumulative HIV new infections prevented over the 10 years will be 46.8% for *S1* (interquartile range [IQR] 32.4%, 60.1%); 29.7% for *S2* (IQR 18.0%, 41.4%), 23.2% for *S3* (IQR 12.2%, 37.0%) and 11.6% for *S4* (IQR 4.0%, 26.6%), respectively. The reproduction number *R*_*0*_ could drop below 1 if there were a substantial reduction of male sexual partners and/or expanded condom use.

**Conclusion:**

Partner reduction is a vital factor within HIV combination interventions to reduce HIV incidence among Beijing MSM, with substantial additional benefits derived from condom use. T&T without substantial partner reduction and increased condom use is less promising unless its implementation were extremely (and improbably) efficient.

## Introduction

China has implemented large and reasonably aggressive nationwide public health programs for HIV prevention and treatment interventions in recent years [1]. These programs have curtailed markedly the spread of HIV through injection drug use (IDU) and contaminated blood/plasma collection and red blood cell reinfusion [2,3]. Mortality among HIV-infected patients has been reduced markedly [2,4,5]. However, no programs have been clearly effective in reversing the rising trend of the HIV epidemic among men who have sex with men (MSM) [6,7]. Based on incidence growth trajectory, MSM have emerged as the most vulnerable group for HIV in China. The proportion of nationally reported new cases of HIV/AIDS attributable to male-to-male sex increased from 0.7% in 2005 [8], to 3.5% in 2007 [9], 10.0% in 2009 [10], 15.0% in 2011 [11], and 21.4% in 2013 [12]. The unrelenting HIV epidemics among MSM in China and elsewhere in the globe demand programmatic innovation, with practical and effective HIV interventions that engage MSM in community partnerships for prevention. One very promising strategy is the Test and Treat (T&T) model [13,14], that combines expanding HIV testing and timely linkage of newly diagnosed individuals to HIV care including risk reduction and antiretroviral therapy (ART) [15]. Prior studies have evaluated individual or collective impacts of T&T intervention components on HIV incidence among MSM [16–22]. We previously published a mathematical model for projecting HIV incidence and prevalence among MSM in Beijing under different scenarios of HIV interventions based on “treatment as prevention” and increased condom use to assess the likely factors with the largest impact on future HIV incidence [23]. However, our model did not have parameter information regarding sexual partner numbers as a parameter. Having unprotected anal intercourse (UAI) with multiple male sex partners has long been recognized as a key element for HIV spread among MSM [19,24–26]. To estimate the interactive effects on the HIV epidemic of three factors—antiretroviral therapy (ART) use behavior, number of male sexual partners, and condom use—we developed a new model to estimate to what extent new HIV infections can be averted by enhanced HIV T&T interventions among MSM in China’s capital city, Beijing, taking partner numbers and condom use into account. This new model took advantage of parameter estimation made possible by 2014 data from a Beijing clinical trial supported under the U.S. National Institutes of Health *Methods in Prevention Practices Program* (MP-3).

## Methods

### Background to social and sexual networks

Social networks have been studied for decades and social network analysis offers important insights into how to conceptualize and model social interactions [27–31]. In physics, networks have been characterized as "scale-free" if they follow a power law with an exponent between 2 and 3. The "scale-free network" describes the degree distribution as a power law, i.e., the probability of having ***k*** partners. ***P*(*k*)** is directly proportional to *k* to the minus ***γ*** [27–31]:

$$P(k)\propto k^{-\gamma }$$

Plotted on a log-log scale, ***γ*** is the slope of the degree distribution, which is a straight line. Scale-free networks have small-world properties, and often display a high clustering coefficient. While scale-free networks are characterized by a power-law decay of the degree distribution ***P*(*k*)** ∝ ***k***^−***γ***^, its cumulative distribution still follows a power-law decay of ***P*(*k*)** ∝ ***k***^−(***γ***−**1**)^.

Formation of sexual networks is similar to other social networks. While most people may have 1–4 sexual partners in a lifetime, some have a much larger number of sexual partners [32,33]. Within an MSM community in a given geographic location, individuals with multiple sexual partners form sexual networks; relationships typically form among individuals with similar attributes, such as age, race or ethnicity, educational achievement, and religious background [33]. A 1996 Swedish sexual behavior survey showed that the cumulative distribution of the lifetime number of sexual partners decays as a scale-free power law (heavy tail distribution), which has a similar exponent for males and females [28,32]. Another analysis of four datasets including two National Surveys of Sexual Attitudes and Lifestyles (NATSAL) in Britain, one sexual behavior survey in rural Zimbabwe, and one survey among MSM in London, showed that all four networks of sexual contacts could be described by power laws over a number of orders of magnitude, and that the derived exponents significantly and meaningfully differed, with an "accelerating network" formed between MSM, in contrast to the heterosexually predominant populations [34].

The web of sexual contacts that has a scale-free structure has numerous interesting implications in terms of disease persistence and intervention policy. Models suggest that: (1) disease control programs are best targeted toward the most sexually active individuals, in accord with most current public health strategies [35]; (2) the HIV/AIDS epidemic will continue to expand and persist even in the case of very small probabilities of transmission per coital episode [34].

### Empirical data used for assumptions and parameter estimations

**Fig 1:**
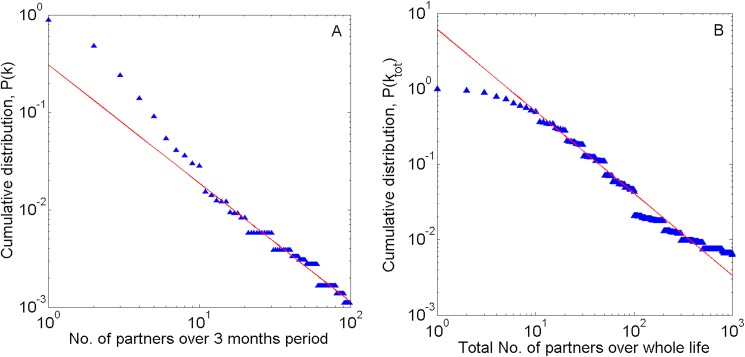
Scale-free distribution of the number of male sexual partners among MSM in Beijing, China. The linear distributions in both (A) and (B) indicate scale-free power-law behavior. Fig 1A: Distribution of number of male sexual partners in the past three months (***k***). The power-law decay index ***γ***−1 = 1.217 (95% CI: 1.174, 1.259), in the range ***k*** ≥10. Fig 1B: Distribution of the total number of male sexual partners in lifetime (***k***_***tot***_). The power-law decay index ***γ***_***tot***_ − 1 = 0.828 (95% CI: 0.812, 0.843), in the range of ***k***_***tot***_ ≥10.

To mathematically characterize the network of sexual partners among Chinese MSM, we analyzed the sexual behaviors of 3588 participants in a cross-sectional survey in Beijing during 2013 and 2014, which was the basis of a clinical trial to test *Multi-component HIV Intervention Packages for Chinese Men Who Have Sex with Men* (the China-MP3 study). To explore whether the networks are scale-free, we explored the tail of the cumulative distribution. In the survey data (number of male sexual partners in the past three months and in a lifetime), the linear regions were both considered to begin at 10 reported partners. We found that the cumulative distribution of the number of sexual partners in the past three months and in a lifetime both decayed as scale-free power laws (Fig 1). Fig 1(A) shows the distribution of the number of sexual partners in the past three months (***k***) with index ***γ*** *(****γ***−1 = 1.217, 95% confidence interval [*CI*]: 1.174, 1.259) in the range of ***k*** *≥* 10. Fig 1(B) shows the distribution of the total number of partners in lifetime (***k***_***tot***_) with index ***γ***_***tot***_ = 1.828 (***γ***_***tot***_ − 1 = 0.828, 95% *CI*: 0.812, 0.843) in the range of ***k*** ≥ 10. In both cases, the power law model provided a good fit to the tail of the distribution.

While the power law model provided a good fit for the available data, the range of number of sexual partners for which the model can be compared with data are limited by our study sample size (***N*** = 3588). The number of orders of magnitude for which data are available reflects the extent to which the scale-free behavior can be confidently attributed to the system. The scale-free region for the lifetime number of sexual partners covers three orders of magnitude. Even with our sample size, this range confirms that there is a large proportion of MSM with many sexual partners. The scale-free nature of the web of sexual contacts among Chinese MSM indicates that intervention programs for reducing sexual partners can prevent HIV spread.

### Mathematical model

HIV transmission in sexual contact networks cannot be well captured by standard men-field models. The complex network model does not have this shortcoming [36–39]. In a network model, individual members constitute nodes, each node with its own degree, such as the number of sexual contacts with other members in gay community; an undirected network of size ***N*** with node degree distribution ***p***(***k***) is thereby obtained. The value $<k>=\sum_{k}p(k)$ is the average number of contacts per member. It was assumed that the population was divided into ***n*** distinct groups of sizes ***N***_***k***_ (***k*** = 1,2,…,***n***) such that each individual in group ***k*** has exactly ***k*** contacts, where ***n*** denotes the maximum degree values of all nodes. If the total population size is $N(N=\sum N_{k})$, then the probability that a uniformly chosen individual has ***k*** contacts is ***p***(***k***) = ***N***_***k***_ / ***N***. The literature has described the transmission of a transmissible disease as a complex network [29–31, 36–39], but the variation of population size over time has been often ignored. In reality, for an infectious disease with a long epidemic course, demographic factors like birth, death, and migration can change the degree distribution ***p***(***k***), and hence the degree distribution is time dependent. Considering that migration is a significant characteristic in the Beijing MSM population, we incorporated the demography of the population into the modeling of HIV epidemic in a complex sexual network context [40–42].

We used a compartmental ordinary differential equations model based on a complex sexual network for simulating and projecting the HIV epidemic with and without enhanced T&T, partner reduction, and condom use interventions among MSM in Beijing. A complex network model rather than a standard epidemiological model was chosen, since the latter is overly simplistic, largely disregarding the complex patterns and structures of sexual contacts with multiple partners. We only considered HIV acquisition to be through homosexual contact with male partners only [43–45]. We think this to be a valid simplification as Chinese MSM are unlikely to acquire HIV from their low-risk female sexual partners [23,46,47], or from sharing needles, since in Beijing, MSM very rarely inject drugs [48,49]. We considered births, deaths, and migration of Beijing MSM in the model, and assumed that their sexually active age was from 15 to 64 years.

**Fig 2:**
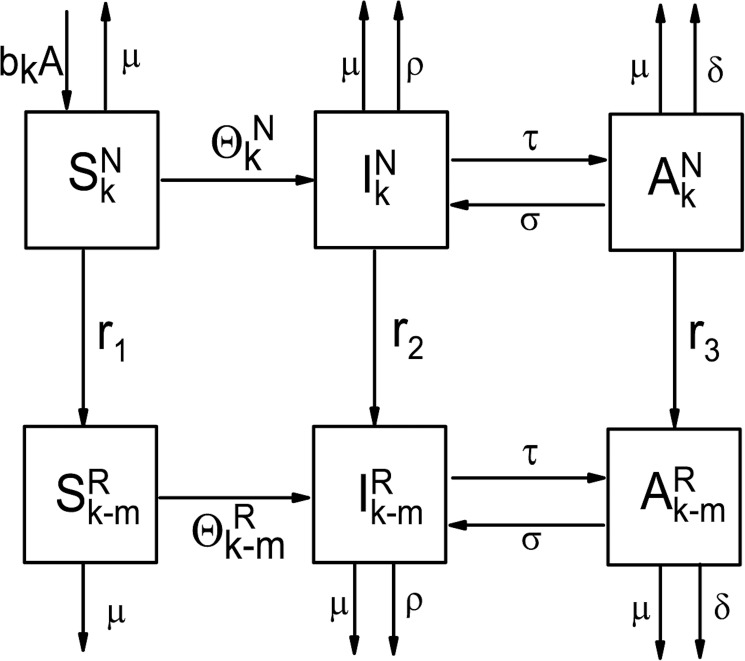
The schematic diagram of the complex network model. The population of MSM in Beijing was divided into two main groups: non-T&T MSM who receive standard of care (compartments in the top row) and T&T MSM who receive enhanced T&T interventions (compartments in the bottom row). It was supposed that the enhanced T&T interventions beyond the standard of care could reduce the number of male sexual partners by (***k***−***m***).

As T&T interventions have correlated with a reduction in risky sexual behaviors [6,50,51], particularly the number of sexual partners, we divided the target population Beijing MSM into two main groups who would receive enhanced T&T interventions (T&T) and who would only receive a current level of standard of care (non-T&T) in the projection time period. Each group was further divided into subgroups of Susceptible (*S*), Infected (*I*), and ART (*A*) (Fig 2). Uninfected MSM who have *k* sexual partners per year enter the model as Susceptible (*S*_*k*_^*N*^), and these men could be local Beijing residents who become 15 years or older, or immigrants from other areas to Beijing. Upon infection, they move from the Susceptible subgroups (*S*_*k*_^*N*^ / *S*_*k*_^*R*^) into Infected subgroups (*I*_*k*_^*N*^/ *I*_*k*_^*R*^), and further to ART subgroups (*A*_*k*_^*N*^ / *A*_*k*_^*R*^) when they initiate ART. The expressions of these subgroups denote both the status of HIV infection and care as well as the size of the population in these compartments at the current time. The superscripts *N* and *R* refer to the two main groups of non-T&T and T&T MSM. For example, *I*_*k*_^*R*^ represents MSM subgroups who are infected HIV and receive T&T intervention, and have ***k*** different male sexual partners per year. It was assumed that a man who received T&T intervention would reduce number of sexual partners by *m*, where m is 0, 1… *n −* 1; and it was also assumed that, when ***k*** ≤***m***,***k***−***m*** = 1, the intervention would help a man reduce his sexual partner to 1 when he has ≤***m*** sexual partners.

### Parameter estimates for the model

There are 13 parameters in the model, including 8 general parameters and 5 enhanced T&T intervention parameters (definitions and uncertainty value ranges in Table 1). These 13 parameters were treated as random variables with a corresponding probability density function. In simulations, 1,000 parameter sets of all 13 parameters were randomly sampled from the corresponding uncertainty value ranges. An asymmetric triangular probability distribution was selected using the Delphi method for these parameters whose most likely value (peak) and range (minimum and maximum values) could be estimated [52,53]; a uniform distribution was selected using the Delphi method (i.e., consensus of informed experts) for other variables when only their range could be estimated [53]. For each uncertainty analysis we used our model along with Latin hypercube sampling (LHS), a type of stratified Monte Carlo sampling [54]. To make predictions, we assigned each parameter a probability density function, which reflects the uncertainty in the possible values of a parameter.

**Table 1:** Parameters and value ranges in the model.

Parameters	Minimum value	Peak value	Maximum value	References or assumption
**General parameters**				
***A***: number of new recruits into Beijing MSM each year †	311527*2%		311527*6.4%	[39, 48–50]
μ: removal rate of Beijing MSM each year (/year) ‡	0.029	0.033	0.04	[46]
***ρ***: disease progression rate without ART, (/year)	1/18	1/12	1/6	[13, 46]
***δ***: disease progression rate on ART, (/year)	1/36	1/24	1/18	[46]
***ε***: ART effectiveness ‡	0.01		0.50	[46]
***β***_***N***_: HIV infectivity without enhanced TNT interventions ‡	0.05		0.15	[46]
***τ***: ART coverage rate ‡	0.475		0.90	Beijing CDC
***σ***: ART quit rate ‡	0.01	0.05	0.25	[46]
**Enhanced TNT intervention parameters**				
***r***_1_: increased coverage of enhanced TNT interventions ‡	0		0.25	Assumed
***r***_2_: increased coverage of interventions among HIV+ ‡	0		0.5	Assumed
***r***_3_: increased ART coverage rate ‡	0		0.5	Assumed
***m***: partner reduction (/year) #	0		50	Assumed
***p***: risk constant ‡	0.2	1.0	1.8	Assumed
***β***_***R***_: HIV infectivity with enhanced TNT interventions ‡				= p * ßN

† These is the estimated number of MSM

‡ These are proportions, and do not have units

# This is the number of sexual partners.

The model makes projections in 10 years since 2013, when there were an estimated 311,557 sexually active MSM living in Beijing [55]. The baseline HIV prevalence among Beijing MSM is 12.7%, based on our cross-sectional survey among 3,588 participants during 2013 and 2014 (our unpublished data). The annual recruitment rate into the target population is from 2.0% to 6.4% [56–59] (Table 1). Both the entrance rate ***b***(***k***) into the sexually active pool of MSM in Beijing and the initial population size in each subgroup follows the degree distribution ***p***(***k***) = 0.15***k***^−2.217^ as confirmed among our China-MP3 study participants. The median duration from infection to progression to AIDS is set at 10 to 12 years for HIV-infected individuals without treatment [13,60,61], and is estimated to be 24 years for those on ART, applying average ART adherence rates.

We assumed that the effectiveness of enhanced T&T interventions was denoted by two indexes: the reduction in the number of male sexual partners (denoted by parameter ***m***) and the infectivity of each HIV-infected man who receives enhanced T&T intervention (denoted by parameter ***β***_***R***_, with ***β***_***R***_ = ***pβ***_***N***_, where ***β***_***N***_ is HIV infectivity among those who do not receive enhanced T&T intervention and *p* is the risk constant). Here we assumed that *m* ranges from 1 to 50, and ***p*** is a risk constant denoting condom use; ***p*** > 1 means reduced condom use and ***p*** < 1 means increased condom use. Therefore, HIV infectivity for enhanced T&T interventions (***β***_***R***_) could be greater or smaller than that for non-T&T (***β***_***N***_). ART effectiveness is defined as HIV infectiousness among HIV-infected MSM who receive ART compared with those who do not receive ART. The estimates for other parameters are showed in Table 1.

#### Scenarios for prediction

We predicted how many new HIV infections could be avoided for the next 10 years from 2014 to 2023 under four different scenarios shown in Table 2. *Scenario 1 (S1)*: With enhanced T&T interventions, the number of male sexual partners decreases (reduced by a random value ***m*** from 1–50) while condom use increases (or risk constant ***p*** is randomly selected between 0.2 and 1]); *S2*: The number of sexual partners decreases (***m*** in [1, 50]) while condom use also decreases (***p*** in [1, 1.8]), e.g., men may be less likely to use condoms with their few stable sexual partners; *S3*: With limited T&T interventions, the number of sexual partners is reduced (***m*** in [1, 10]) while condom use may increase or decrease (***p*** in [0.2, 1.8]); *S4*: T&T interventions only has an impact on MSM with ≥100 male sexual partners, including reducing sexual partners (***m*** in [1, 50]) and increasing condom use (***p*** in [0.2, 1]).

**Table 2:** Scenarios for prediction.

Scenario 1	Scenario 2	Scenario 3	Scenario 4 (only applicable to participants with sexual partners >100)
***m*** ∈ [1, 50]	***m*** ∈ [1, 50]	***m*** ∈ [1, 10]	***m*** ∈ [1, 50]
***p*** ∈ [0.2, 1]	***p*** ∈ [1, 1.8]	***p*** ∈ [0.2, 1.8]	***p*** ∈ [0.2, 1]

#### Time-dependent and extensive sensitivity analyses

To identify the key factors that determine the impact of enhanced T&T interventions, we performed time-dependent sensitivity analyses [54, 62]. We used the 10 years of predicted data from the uncertainty analyses to calculate time-dependent sensitivity coefficients; a partial rank correlation coefficient (PRCC) was calculated annually for each parameter. Table 3 shows the key factors for determining the impacts of enhanced T&T intervention on preventing HIV new infections in years 1, 5, and 10, under varying scenarios.

**Table 3:** Time-dependent sensitivity coefficients (PRCCs) for key parameters: impacts on preventing HIV new infections.

Key parameters	Scenario 1 (S1)			Scenario 2 (S2)		
	Year 1	Year 5	Year 10	Year 1	Year 5	Year10
Increased coverage rate (***r***_1_),%	0.929	0.954	0.894	0.714	0.847	0.668
Partner reduction (***m***)	0.674	0.650	0.614	0.798	0.671	0.643
Increased ART coverage rate (***r***_3_),%	0.832	0.513	0.443	0.756	0.433	0.454
Risk constant (***p***)	-0.560	-0.571	-0.619	-0.304	-0.355	-0.463
ART quit rate (***δ***)	-0.107	-0.291	-0.493	-0.116	-0.252	-0.527
Transmission rate (***β***_***N***_)	-0.023	-0.453	-0.595	-0.134	-0.406	-0.635

Note: MSM: men who have sex with men; ART: antiretroviral therapy.

To assess how robust our results were, given uncertain parameter estimations, we also ran extensive sensitivity analyses to assess the influence of each parameter on thresholds of this model. A total of 1,000 parameter sets of all 13 parameters were randomly sampled from their corresponding uncertainty ranges (Table 1). We treated the 13 parameters as random variables with a corresponding probability density function; samples were then taken from these probability density functions, thus deriving the frequency distributions for the basic reproductive number (***R***_0_). ***R***_0_ is the number of secondary cases produced by a typical HIV-infected man during his entire period of infectiousness in a demographically steady susceptible MSM population. Calculating ***R***_0_ is critical to determine whether HIV will increase, stabilize, or decline among the MSM population in Beijing. For this model, we calculated the basic reproduction number ***R***_0_ by the method of van den Driessche and Watmough [63].

## Results

### Predictions and uncertainty estimations

**Fig 3:**
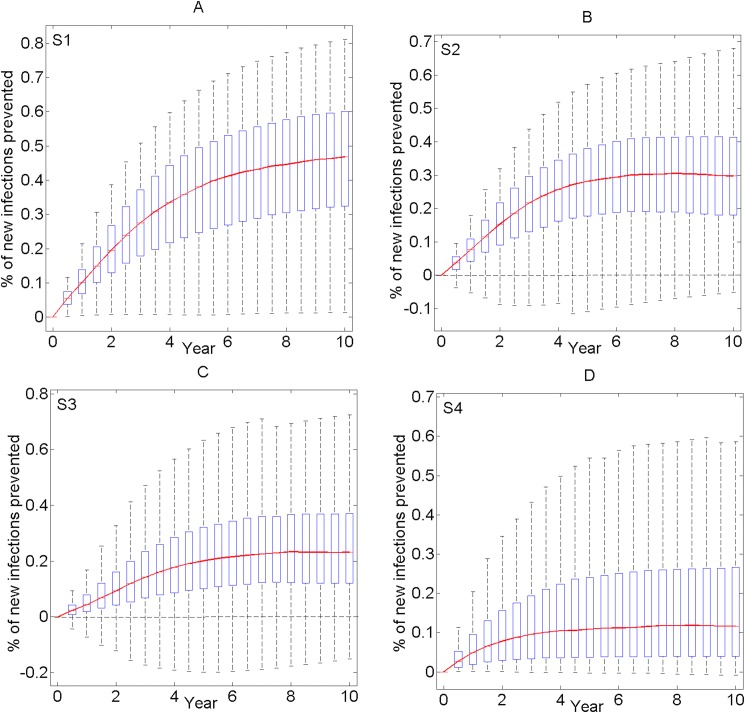
Projections of cumulative HIV new infections prevented and time-dependent uncertainty analyses of enhanced T&T interventions. Each box-plot represents the results of 1000 simulations for every 6 months. These plots show median values (horizontal red lines), upper and lower quartiles (blue boxes), and outlier cutoffs (dashed lines).

Assuming that the baseline levels of HIV care prevalent in 2013 are sustained, HIV prevalence among Beijing MSM will increase rapidly until it reaches a plateau level of 23.2% (median value) by 2023. Under scenarios 1–4 of enhanced T&T interventions, the cumulative HIV new infections prevented over the next 10 years will be 46.8%, 29.7%, 23.2% and 11.6%, respectively (Fig 3).

The 1000 sets of simulations for Scenario 1 showed that the interquartile ranges (IQR: 25%-75%) of HIV infections prevented by the tenth year are from 32.4%-60.1%, and the finite outliers are 1.3% and 81.2% (Fig 3A). The simulations for other scenarios showed:

- Scenario 2: IQR: 18.0%-41.4%; outliers: 0 to +68.1% (Fig 3B);
- Scenario 3: IQR: 12.2%-37.0%; outliers: -15.1% to +76.7% (Fig 3C);
- Scenario 4: IQR: 4.0%-26.6%; outliers: -0.8% to +70.9% (Fig 3D).

### Time-dependent analyses

Table 3 shows the relative importance of six parameters in the model in predicting HIV new infections averted. The top four factors that determine the impacts of enhanced T&T interventions are the coverage rate of interventions among HIV-uninfected MSM (***r***_1_), ART coverage rate (***r***_3_), partner reduction (***m***), and a risk constant (***p***), where ***r***_1_ is the most important factor during the entire prediction period under Scenario 1, and after year 1 under Scenario 2.

**Fig 4:**
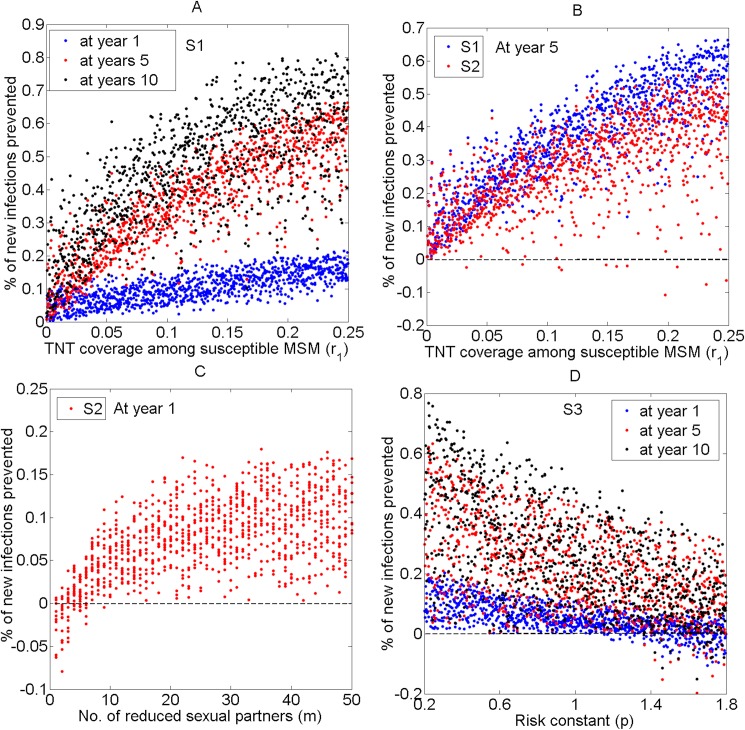
Uncertainty analyses of unadjusted data from Scenarios 1–3: each dot represents one simulation. (A) Scatterplot showing the impacts of enhanced T&T coverage rate among susceptible MSM (***r***_1_) on averted HIV new infections in 1 (blue dots), 5 (red dots), and 10 years (black dots) under Scenario 1. (B) Scatterplot showing the impacts of T&T coverage rate among susceptible MSM (***r***_1_) on averted HIV new infections in 5 years under Scenario 1 (blue dots) and Scenario 2 (red dots). (C) Scatterplot showing the impacts of partner reduction (***m***) on averted HIV new infection in 1 year under Scenario 2. (D) Scatterplot showing the impacts of risk constant (***p***) on averted HIV new infections in 1(blue dots), 5 (red dots), and 10 years (black dots) under Scenario 3.

Increasing the coverage rate of enhanced T&T interventions among susceptible MSM will prevent a substantial number of HIV new infections under Scenario 1 (Fig 4A) and even under Scenario 2 (Fig 4B). The number of averted infections will increase over time (Fig 4A). Only in very few simulations under Scenario 2, the impact may be negative due to risk compensation, i.e., a decrease in condom use (dots under 0; Fig 4B).

The impact of partner reduction (***m***) under Scenario 2 is influenced by condom use (or risk constant ***p***). The benefit of reducing sexual partners by < 10 is easily offset by reduced condom use (Fig 4C). Scatter plots of partial rank correlations, strong or weak, of parameters ***r***_1_ and ***m*** in Fig 4B and 4C, also reflect the order of importance (Table 3). Under Scenario 3, risk proportion (***p***) and T&T coverage rate among susceptible MSM (***r***_1_) are the top two factors that influence the impact of the interventions. The partial rank correlation coefficients for parameter ***p*** in 1, 5, and 10 years are −0.853, −0.805 and −0.832, respectively. The benefit of reducing sexual partners under Scenario 3 is largely offset by either increased risk behavior or decreased condom use (Fig 4D).

### Extensive sensitivity analyses

**Fig 5:**
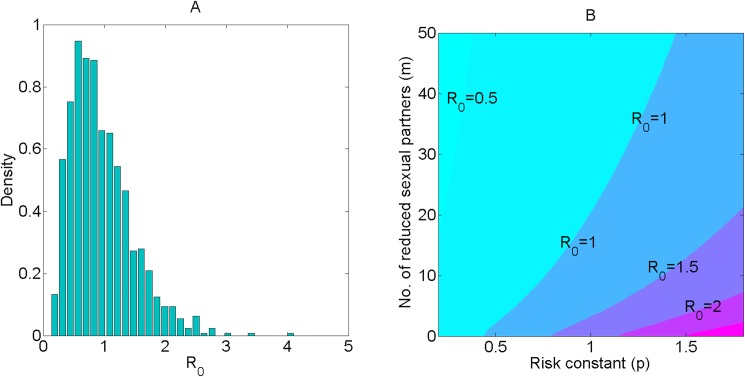
**Uncertain analysis (A) and sensitive analysis (B) of reproduction number *R***_0_**.** (A) Distribution of ***R***_0_ values obtained from Latin Hypercube Sampling for 13 input parameters as shown in Table 1, with 1000 simulations; (B) Distribution of ***R***_0_ values due to partner reduction (***m***) and change of risk constant (***p***).

Under the uncertainty ranges of eight universal parameters and no enhanced T&T interventions (five T&T parameters set as zero; Table 1), the distribution of the basic reproduction number *R*_*0*_ has a mean of 1.67 (standard deviation [*SD*]: 0.68; 95% confidence interval [*CI*], 0.35–2.99; interquartile range [*IQR*]: 1.16–2.09). When five T&T parameters were set as >0, the distribution of ***R***_0_ has a mean of 0.96 (*SD*: 0.53; 95% *CI*, 0.02, 2.00; *IQR*: 0.60–1.22; Table 1). Fig 5A shows ***R***_0_ distribution under a variety of coverage rates of enhanced T&T interventions, where the x-axis shows the value of ***R***_0_ and the y-axis shows the value of ***R***_0_ probability density. Fig 5B shows ***R***_0_ distribution in response to a range of partner reduction (***m***) and risk constant (***p***) while other parameters were set as their peak or mean values. It shows that ***R***_0_ has a good chance to fall to < 1, even if MSM increase their other risk behaviors, but only if there were to be a substantial reduction of male sexual partners.

## Discussion

MSM continue to be disproportionately affected by HIV since the first cases emerged in the 1970s [64]. The driving force of transmission is a combination of unprotected anal sex (especially receptive) and multiple at-risk male sexual partners. Same-sex marriage is not recognized in China and many MSM have a large number of lifetime sexual partners, either sequentially or concurrently [65,66]. Our model sends a cautionary note to the global HIV prevention community since it suggests that universal T&T will not reduce MSM HIV prevalence if MSM do not also reduce risky behaviors, unless programs and communities can achieve improbably high T&T penetrance and intervention fidelity. If coverage with T&T rises, it could promote community attitudes that are positive as to awareness of HIV status, fostering earlier initiation of ART. Then if MSM are retained in care and adherent to ART, their viral loads are reduced and their infectivity to others is diminished, the “treatment as prevention” paradigm. Persons aspiring to World Health Organization “90-90-90” coverage goals of testing, linkage, and viral-suppressive therapy, respectively, often emphasize the biomedical aspect of T&T, e.g. ART and reduction of viral load, but do not acknowledge the mathematical ease with which the public health benefits of therapy can be overcome with high risk behaviors and suboptimal ART adherence. Our model reinforces the urgency with which HIV-infected MSM must be linked to programs including ***both*** ART and risk reduction behavioral interventions. Our model is quite convincing that reducing male sexual partner numbers is a crucial element of combination HIV interventions for preventing new infections among Beijing MSM, and HIV transmission trends are likely to decline only as long as there is a substantial reduction of male sexual partners, even when MSM do not increase condom use with their fewer sexual partners. Partner reduction could offset the impact of decreased condom use and prevent a substantial number of new infections. In turn, increased condom use can contribute substantially towards reduced HIV incidence, though not to the degree of partner reduction.

Since the literature has suggested that ART may have varying impacts on the HIV epidemic [13,54,67,68], we assessed its impact under a range of assumptions in detailed sensitivity analyses of T&T and ART parameters (Fig 4C). For other sensitive analyses, we supposed the impact of ART to be the average of all these possible values.

A few studies have modeled the impact of T&T interventions on the HIV epidemics among Chinese MSM, and the predicted results have varied [23,69,70]. One model concluded that early initiation of ART among MSM across the country may or may not reduce the number of new HIV infections in the settings where the availability of effective drugs is limited [69]. Two models focusing on MSM in Beijing suggested the benefits of T&T [23,70], but one was conditional on significant increases in condom use [23]. These existing models did not include the number of sexual partners as a risk parameter. Sexual networks must be considered in models predicting new HIV infections averted under difference scenarios of sexual behavioral change; the cumulative distribution of the number of sexual partners complied with the scale-free power laws that we derived using data from our large community-based survey among Beijing MSM [71,72]. While mathematical modeling can be used to guide public health decision making [73], it is only a projection under varying assumptions, not a true mirror of reality. Our model reflects current biomedical understanding and seeks to balance parsimony of the model parameters and reality. We tried to capture the possible processes of the dynamics of HIV transmission under enhanced T&T interventions among MSM, using recently refined parameters that are specific for Beijing. If pre-exposure prophylaxis with antiretroviral drugs were to become available to Chinese MSM, and if uptake and adherence were substantial, then our real world model will need expansion with data-derived parameter estimations to assess this new tool.

Our model may also be helpful for forecasting and evaluating HIV intervention programs in other MSM communities; it is likely that other venues, too, will note HIV incidence to be highly sensitive to partner reduction as the principal determinant. While varying penetration of ART and condom use influenced the models, the dominant variable was the number of sexual partners. These findings have implications for a variety of HIV prevention activities targeting MSM population, including community engagement, health education, peer counseling, behavioral interventions, and, sometimes, engagement of women [74]. Internet use is increasingly common and smart phones are universally available among urban Chinese MSM. Internet hook-up sites facilitate finding new male sexual partners, as with men’s use of Chinese social media (e.g., QQ, WeChat, and Grinder) [75]. At the same time, social media can be used to deliver HIV prevention interventions [76–79] anchored on promoting safer sex through stable sexual partnerships and condom use. This combination prevention research agenda towards social marketing, incentive-based behavior change, and community engagement to both reduce high risk behavior and to engage antiretroviral therapy or prophylaxis is every bit as urgent as the more unitary biomedical/pharmacological approaches [15,23,57,80–84].
